# Research Progress on Chinese Herbal Medicine Components Targeting Ferroptosis for Cancer Therapy

**DOI:** 10.3390/molecules31121985

**Published:** 2026-06-06

**Authors:** Nanhao Zhou, Yuansheng Zhang, Chenyu Wang, Xianbo Mou

**Affiliations:** Health Science Center, Ningbo University, Ningbo 315211, China; 226002158@nbu.edu.cn (N.Z.); 226002142@nbu.edu.cn (Y.Z.);

**Keywords:** traditional Chinese medicine, ferroptosis, gastrointestinal malignancies, respiratory tumors, osteosarcoma, breast cancer, hematological malignancies, glioblastoma

## Abstract

Recent studies indicate that ferroptosis shows unique advantages in oncotherapy, particularly in reversing multidrug resistance (MDR). Despite current therapeutic advancements, the treatment of high-incidence malignancies with dismal prognoses continues to face challenges, including limited clinical efficacy, significant side effects, and drug resistance. In recent years, Chinese herbal medicine (CHM) has gained increasing attention in anti-tumor therapy. CHM bioactive components are highly effective in inducing tumor cell ferroptosis, inhibiting tumor proliferation and migration, and reversing drug resistance. Additionally, some components can protect normal cells and improve the tumor microenvironment. This review systematically summarizes the regulatory roles of various CHM bioactive components in ferroptosis across common human cancers. We further analyze the underlying molecular mechanisms, focusing on the modulation of key regulatory targets (e.g., GPX4, SLC7A11, and Nrf2) and critical signaling cascades (e.g., PI3K/AKT/mTOR and p53). Furthermore, the differential effects of bioactive compounds from CHM on common tumors were evaluated, alongside their potential in combination therapy. This review provides a theoretical foundation for the development of novel anticancer drugs targeting ferroptosis regulation and offers new perspectives for the clinical application of CHM in oncology.

## 1. Introduction

Chinese herbal medicine (CHM) represents a distinctive therapeutic approach in China and serves as an indispensable component of comprehensive cancer therapy. The clinical efficacy and therapeutic roles of CHM in oncology have garnered increasing recognition and validation from both the public and medical communities. Particularly for highly prevalent malignancies with poor prognoses, conventional therapeutic modalities such as surgery and chemotherapy are frequently hindered by limited efficacy and pronounced toxic side effects [1]. In stark contrast, CHM offers distinct advantages, including high affordability and lower rates of acquired drug resistance and tumor recurrence [2]. Driven by continuous advancements in modern pharmacology and nanotechnology, numerous CHM bioactive components have gained considerable attention and are being applied as cancer therapeutics. Consequently, their antitumor mechanisms are progressively being elucidated. For instance, CHM-derived extracellular vesicles can modulate the immunosuppressive multifaceted targeting of tumor microenvironment, thereby activating immune responses and enhancing overall antitumor immunity [3]. However, the tumor microenvironment (TME) serves as the central factor dictating the outcomes of immunotherapy. Although the host immune system can typically identify and eliminate tumor cells, tumors actively suppress anti-tumor immune responses through direct and indirect mechanisms, fostering an immunosuppressive microenvironment defined by hypoxia, hypertonicity, acidity, and an abundance of inflammatory mediators [4]. Furthermore, polyphenol nanoparticles exhibit remarkable cancer-cell-targeting capabilities and serve as robust drug delivery platforms, demonstrating substantial therapeutic potential in oncology [5].

Among various mechanisms, ferroptosis is a novel form of programmed cell death distinct from apoptosis and autophagy. Its core features are the iron-dependent accumulation of lipid peroxides and the functional inactivation of antioxidant systems, such as glutathione peroxidase 4 (GPX4), ultimately leading to cell membrane disintegration [6,7]. Since its discovery, this unique regulatory mechanism has rapidly emerged as an interdisciplinary research focus in the life sciences and medical fields. Ferroptosis plays a pivotal role in the pathological progression of various diseases, such as tumors, metabolic disorders, and neurodegenerative diseases, thereby providing novel therapeutic targets and directions [8]. In the field of oncology, regulatory strategies targeting key molecules, such as acyl-CoA synthetase long-chain family member 4 (ACSL4) and solute carrier family 7 member 11 (SLC7A11), offer breakthrough solutions for drug-resistant malignancies like colorectal cancer and nasopharyngeal cancer [9,10]. Concurrently, a substantial body of evidence indicates that the induction of ferroptosis can effectively inhibit tumor cell proliferation and migration [11]. Furthermore, the combined application of ferroptosis agonists with chemotherapy, radiotherapy, and immunotherapy has exhibited remarkable synergistic efficacy in preclinical studies of breast, ovarian, and other cancers [12,13,14].

In recent years, alongside deepening research, accumulating evidence indicates that bioactive components of CHM exert anticancer effects by inducing ferroptosis in tumor cells [15]. This discovery not only highlights the immense potential of CHM in oncology but also provides novel insights for the development of emerging anticancer drugs and the exploration of unknown therapeutic targets. Integrating current research progress, CHM bioactive components have demonstrated potent ferroptosis-regulatory capabilities across multiple highly prevalent clinical malignancies, including gastrointestinal malignancies, respiratory malignancies, and others [16,17]. These active components primarily trigger intracellular lipid peroxidation and iron accumulation by directly or indirectly modulating key molecules such as GPX4, SLC7A11, Nrf2 (nuclear factor erythroid-2-related factor 2), and p53, while broadly interfering with crucial signaling pathways like PI3K/AKT/mTOR [15,18,19]. Notably, this regulatory effect is prominent not only in inducing ferroptosis and inhibiting tumor proliferation and migration but also demonstrates unique clinical advantages in reversing tumor drug resistance [20]. Based on these premises, this article systematically reviews the regulatory mechanisms and recent research progress regarding ferroptosis induction by CHM bioactive components in common cancers, thereby providing a robust theoretical foundation for the clinical translation and application of CHM in oncology.

## 2. Gastrointestinal Malignancies

In 2022, over 18% of global cancer incidences and more than 33% of cancer-related mortalities were attributed to gastrointestinal (GI) cancers [21]. This category encompasses colorectal, gastric, liver, pancreatic, and esophageal cancers, among which colorectal, gastric, and liver cancers exhibit the highest incidence rates and remain the primary focus for clinical practitioners and researchers. Currently, clinical management of GI malignancies predominantly relies on chemotherapy and surgical resection. However, these approaches are frequently hindered by limited therapeutic efficacy and dismal prognoses. Accumulating evidence suggests that the induction of ferroptosis can effectively suppress the proliferation and migration of GI tumor cells and reverse MDR [22]. With the increasing prominence of CHM, an increasing number of scholars have begun to recognize the remarkable role of CHM bioactive compounds in ferroptosis induction and the treatment of GI cancers.

### 2.1. Colorectal Cancer

The global incidence and mortality of colorectal cancer (CRC) rank third and second, respectively, identifying it as the most prevalent malignancy among gastrointestinal (GI) tumors [21].

**Figure 1:**
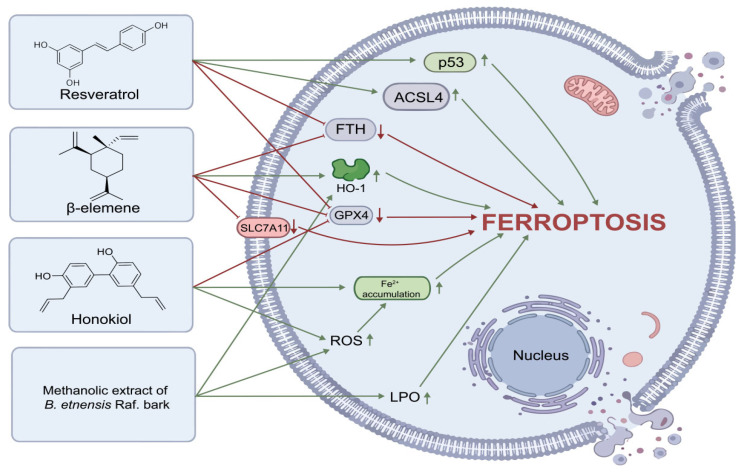
Schematic representation of CHM bioactive compounds and extracts inducing ferroptosis in CRC cells. CHM bioactive components inhibit CRC by targeting distinct ferroptotic pathways. Specifically, resveratrol upregulates ACSL4 and p53 while downregulating GPX4 and FTH. β-elemene suppresses GPX4, FTH, and SLC7A11 alongside HO-1 induction. Honokiol inhibits GPX4, driving the accumulation of ROS and intracellular Fe^2+^. Furthermore, the methanolic extract of *B. etnensis* Raf. bark elevates LPO and ROS levels, accompanied by HO-1 upregulation.

It has been demonstrated that in CRC cells treated with resveratrol, the expression levels of ACSL4, which promotes cell ferroptosis, and p53 protein increased, alongside the downregulation of anti-ferroptotic proteins such as GPX4 and ferritin heavy chain (FTH) [23]. These findings validate that resveratrol exerts its anti-tumor effects on CRC via the ferroptosis pathway [23]. Additionally, the combined application of turmeric extract β-elemene and cetuximab has been shown to induce ferroptosis and inhibit metastasis in colorectal cancer cells through multiple mechanisms [24]. Honokiol, isolated from Magnolia grandiflora, triggers ferroptosis in CRC cells by attenuating GPX4 activity and augmenting the accumulation of reactive oxygen species (ROS) and ferrous ions (Fe^2+^) [25]. Furthermore, studies have demonstrated that the methanolic extract of *Betula etnensis* Raf. bark elevates the levels of lipid peroxidation (LPO) and ROS in Caco-2 human colon cancer cells. It also significantly upregulates the expression of heme oxygenase-1 (HO-1), leading to a marked inhibition of cell viability [26] (Figure 1). A summary of these CHM bioactive components against colorectal cancer is presented in Table 1.

### 2.2. Gastric Cancer

The global incidence and mortality of gastric cancer (GC) rank fifth overall, making it the second most prevalent gastrointestinal malignancy after colorectal cancer [21]. Honokiol, as previously mentioned, exhibits significant therapeutic potential in GC management. Experimental evidence indicates that honokiol suppresses the growth of GC cells and induces ferroptosis, a process potentially mediated by the inhibition of Yes-associated protein (YAP) signaling [27]. Ophiopogonin B has been shown to downregulate the expression of GPX4 and SLC7A11. Compared to the control group, Ophiopogonin B significantly reduced the volume and weight of tumors in AGS cell-derived xenograft models, confirming that it triggers ferroptosis in GC cells by disrupting the GPX4/SLC7A11 axis [28]. Curcumin has similar effects to ophiopogonin B in GC cells. In addition, it inactivates the PI3K/AKT/mTOR signaling pathway, thereby triggering autophagy-mediated ferroptosis [29]. Both direct and indirect evidence provided by Guan et al. [30] demonstrate that the anti-gastric cancer activity of Tanshinone IIA (Tan IIA) is mediated through ferroptosis induction. Specifically, Tan IIA increases ROS levels and upregulates the ferroptosis markers prostaglandin-endoperoxide synthase 2 (Ptgs2) and ChaC glutathione-specific gamma-glutamylcyclotransferase 1 (Chac1), while simultaneously enhancing p53 expression and suppressing SLC7A11. These findings successfully elucidated partial anticancer mechanisms of Tan IIA. Furthermore, Baicalin, extracted from *Scutellaria baicalensis*, not only regulates the p53/SLC7A11 axis to induce ferroptosis in GC cells [31] but also reverses resistance to the conventional chemotherapeutic agent 5-fluorouracil (5-FU) through ferroptosis-mediated mechanisms, thereby sensitizing cells to its anticancer effects [32]. Similarly, brusatol, potentially through the mechanism of inhibiting the Nrf2/HO-1 signaling pathway, increases the levels of ROS and LPO, as well as the concentration of Fe^3+^ in HGC-27 GC cells, thereby inducing the occurrence of ferroptosis [33]. Additionally, a2, a derivative of the natural product oridonin, can decrease the expression of GPX4 in GC cells and lead to Fe^2+^ accumulation via the autophagy pathway. Meanwhile, in vivo studies found that the anticancer efficacy of a2 is superior to that of 5-FU, demonstrating the immense clinical application value of this novel compound [34]. A summary of these components is provided in Table 2.

### 2.3. Hepatocellular Carcinoma

**Figure 2:**
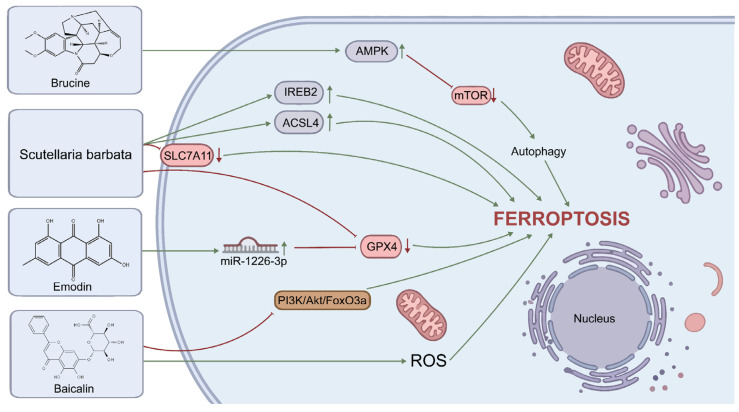
Schematic representation of CHM bioactive compounds and extracts inducing ferroptosis in HCC cells. CHM bioactive components modulate the ferroptosis network in HCC via distinct targets: brucine activates AMPK to inhibit mTOR, thereby triggering autophagy-dependent ferroptosis; *Scutellaria barbata* upregulates IREB2 and ACSL4 while suppressing SLC7A11 and GPX4; emodin upregulates miR-1226-3p to subsequently block GPX4; and baicalin elevates ROS levels while inhibiting the PI3K/Akt/FoxO3a signaling pathway.

The global incidence of hepatocellular carcinoma (HCC) ranks sixth, while its mortality rate has risen to the third highest among all malignancies, making it the second most lethal gastrointestinal cancer after colorectal cancer [21]. Research indicates that brucine can inhibit the growth of HCC cells both in vivo and in vitro, mediating the activation of adenosine monophosphate-activated protein kinase (AMPK) to induce autophagy and regulate ferroptosis in cancer cells [35]. Animal experiments have demonstrated that oral administration of *Scutellaria barbata* can effectively inhibit HCC tumorigenicity, increasing the expression of iron-responsive element binding protein 2 (IREB2) and ACSL4 while reducing the expression of GPX4 and SLC7A11, thereby significantly suppressing HCC growth through the regulation of ferroptosis [36]. In vitro experiments by Zhu [37] found that emodin upregulates miR-1226-3p to subsequently downregulate the expression of GPX4, modulating this axis to induce the occurrence of HCC ferroptosis; in vivo experiments in nude mice proved that emodin can reduce the levels of GPX4 in subcutaneous xenograft tumors, promoting ferroptosis and significantly inhibiting tumor growth. An et al. [38] identified the joint targets of puerarin, ferroptosis, and HCC through bioinformatic analysis. It was concluded that puerarin may inhibit HCC by targeting AKR1C3 (aldo-keto reductase family 1 member C3) to regulate ferroptosis. Although these findings lack experimental validation in cell or animal models, they suggest that puerarin has significant therapeutic potential. Additionally, baicalin induces ferroptosis in HepG2 cells by increasing ROS levels and inhibiting the PI3K/Akt/FoxO3a pathway [39] (Figure 2). This mechanism differs from the one observed in GC, which may be explained by cell-type-specific differences. Furthermore, the effects of CHM bioactive components can vary even across different cell lines of the same cancer. For instance, Formosanin-C (FC) shows stronger ferroptosis-inducing effect in HepG2 cells than in Hep3B cells [40]. This is attributed to the higher expression of nuclear receptor coactivator 4 (NCOA4) and lower expression of FTH1 in HepG2 cells, which collectively enhance the ferroptotic effect. Icariin significantly inhibits the expression levels of GPX4, SLC7A11, peroxisome proliferator-activated receptor gamma (PPARG), and fatty acid-binding protein 4 (FABP4), thereby inducing ferroptosis in HepG2 cells through the PPARG/FABP4/GPX4 pathway [41]. Similarly, cryptotanshinone exhibits strong inhibitory effects on HepG2 cells. Its mechanism involves the downregulation of GPX4 and SLC7A11, which leads to ROS accumulation and ferroptosis [42]. Although in vivo data are lacking, the shared anticancer mechanisms between cryptotanshinone and other drugs suggest its potential for combination therapy. In addition to single components, CHM formulas such as Shugan Quyu Jiedu, Huayu Qutan, Shipi Xiaoji, and Xiaoai Jiedu also show significant value [43]. Specifically, Huayu Qutan Formula protects normal hepatocytes by decreasing p53 and increasing GPX4 and SLC7A11 expression at both mRNA and protein levels [44]. This attenuates lipid deposition and oxidative damage, effectively inhibiting ferroptosis. Unlike conventional drugs that target HCC cells to induce ferroptosis, Huayu Qutan Formula focuses on preventing liver cancer by protecting normal cells. This approach offers a new strategy for future drug development. The detailed information of these components against liver cancer is summarized in Table 3.

### 2.4. Pancreatic Cancer

Although the global incidence of pancreatic cancer is not among the top ten, its mortality rate ranks sixth worldwide, second only to GC [21]. Celastrol (Cel) suppresses the expression of GPX4 by promoting its ubiquitination, which reduces the viability of PANC-1 pancreatic cancer cells. This effect can be reversed by the ferroptosis inhibitor Ferrostatin-1 (Fer-1), demonstrating that Cel exerts its anticancer activity by inducing ferroptosis [45]. Nrf2 is a master regulator of the antioxidant response, and GPX4 and SLC7A11 are its downstream targets [46]. Wogonin decreases Nrf2 expression, subsequently inhibiting GPX4 through the Nrf2/GPX4 axis. This leads to elevated Fe^2+^ levels and lipid peroxidation, ultimately inducing ferroptosis in pancreatic cancer cells [47]. Intervention with Solasonine (SS) in PANC-1 and CFPAC-1 pancreatic cancer cells was found to eliminate their migration and invasion capabilities. SS promotes ferroptosis by inhibiting transcription factor AP-2 alpha (TFAP2A) protein levels. Because the promoter region of the deubiquitinase OTUB1 contains a TFAP2A binding site, SS reduces OTUB1 expression, which in turn activates the ubiquitin-mediated degradation of SLC7A11, thereby promoting ferroptosis in pancreatic cancer cells [48]. Additionally, Cui et al. [49] demonstrated that ponicidin decreases free glutathione (GSH) levels and GPX4 activity. This compound induces ferroptosis in SW1990 pancreatic cancer cells by inhibiting the γ-glutamyl cycle and regulating polyunsaturated fatty acid (PUFA) metabolism. A comprehensive summary of these findings is provided in Table 4.

### 2.5. Esophageal Cancer

Esophageal cancer is a high-incidence malignancy in China with a poor prognosis, predominantly manifesting as esophageal squamous cell carcinoma (ESCC) [50]. Although its global incidence is not within the top ten, its mortality rate ranks seventh worldwide, second only to pancreatic cancer [21]. Ferulic acid (FA) is a CHM bioactive component extracted from various plants. ESCC cells stimulated by FA show significantly reduced colony formation and cell viability. This effect is driven by increased ROS level, enhanced ACSL4 and HO-1 activities, and decreased SLC7A11 and GPX4 levels, resulting in potent anticancer activity [51]. Similarly, trigonelline inhibits the Nrf2/SLC7A11/GPX4 signaling pathway, which elevates ROS and iron levels. This not only suppresses the proliferation and migration of KYSE-150 cells but also promotes ferroptosis in KYSE-150 cells [52]. Furthermore, the pro-oxidant effect of Huqi Powder triggers ROS accumulation, which disrupts cellular redox and mitochondrial homeostasis, thereby upregulating p53 and inducing both mitochondrial apoptosis and ferroptosis in EC9706 cells [53]. *Salvia chinensis* Benth. is a traditional CHM used to treat esophageal cancer. Findings by Lin et al. indicate that intervention with this CHM significantly inhibits the progression of orthotopic esophageal cancer in mice. This process involves increased NCOA4 expression and decreased GPX4 protein levels, which ultimately triggers ferroptosis [54]. Artesunate, a semi-synthetic derivative of artemisinin, suppresses the proliferation and migration of KYSE-150 cells while promoting the accumulation of intracellular ROS and Fe^2+^. The specific mechanism may involve artesunate-induced ferritin degradation, which disrupts cellular iron homeostasis and leads to ferroptosis [55]. Additionally, resveratrol not only exhibits anti-colorectal cancer activity but also shows potential for combination therapy with cisplatin. It may reverse the resistance of the Eca190/DDP cell line by downregulating FTH and GPX4 expression while upregulating ACSL4 and p53 levels [56]. Information regarding these CHM bioactive components is summarized in Table 5.

Although the clinical translation of a specific CHM bioactive component such as cryptotanshinone in gastrointestinal malignancies is currently hindered by a reliance on in vitro cellular assays, some compounds have progressed further. Specifically, components such as brucine and emodin have shown robust antitumor efficacy in in vivo xenograft models [35,37], marking them as more mature preclinical candidates. Nevertheless, the future clinical application of potent alkaloids like brucine requires careful evaluation of their narrow therapeutic window and intrinsic toxicity [57].

## 3. Respiratory Tumors

Respiratory tumors primarily encompass lung cancer and nasopharyngeal carcinoma (NPC). According to Global Cancer Statistics 2022 report, lung cancer is not only a primary constituent of respiratory tumors but also remains the leading malignancy globally in terms of both incidence and mortality. With 2,480,675 new cases annually—accounting for 12.4% of all cancer diagnoses—it poses a severe threat to public health [21]. In contrast, NPC is relatively rare. In 2020, there were approximately 133,354 new cases, representing only 0.7% of all newly diagnosed cancers [58]. Notably, its incidence is significantly higher in Asia than in Western countries, with approximately 85.2% of new cases occurring in Asia. Current management of lung cancer and NPC mainly relies on surgery, chemotherapy, and targeted therapy. However, the efficacy of these conventional modalities is often limited, and associated adverse effects inevitably impair patients’ quality of life [59,60]. Consequently, there is an urgent need for novel therapeutic strategies or agents, positioning CHM bioactive components as a field of growing academic interest with unique therapeutic value.

### 3.1. Lung Cancer

Lung cancer is a malignancy originating from the bronchial or tracheal mucosa or glands, with non-small cell lung cancer (NSCLC) being the primary subtype, accounting for approximately 85% of all cases [61]. As the understanding of ferroptosis continues to deepen, recent advances have highlighted the progression of ferroptosis as an indispensable therapeutic avenue for NSCLC intervention [62]. Consequently, natural products and bioactive components from CHM are being actively explored to capitalize on this targeted mechanism.

**Figure 3:**
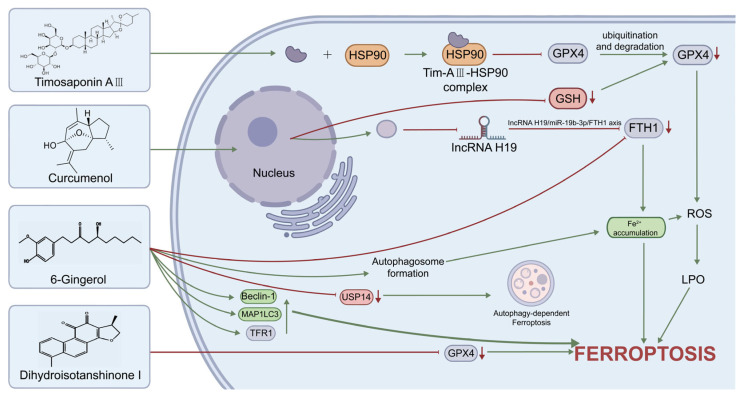
Schematic representation of CHM bioactive compounds inducing ferroptosis in lung cancer cells. CHM bioactive components exert potent anti-tumor efficacy against lung cancer via distinct ferroptosis pathways: Timosaponin A-III binds to HSP90 to form a complex that ubiquitinates and degrades GPX4; curcumenol suppresses lncRNA H19, thereby downregulating FTH1 via the lncRNA H19/miR-19b-3p/FTH1 axis, depleting GSH, and accumulating Fe^2+^ and ROS. 6-gingerol promotes autophagy-dependent ferroptosis by inhibiting USP14, accompanied by the upregulation of Beclin-1, MAP1LC3, and TFR1, the downregulation of FTH1, and the accumulation of Fe^2+^ and ROS. Dihydroisotanshinone I triggers ferroptosis by directly downregulating GPX4. In the schematic, green arrows represent activation, upregulation, or the downstream flow of signaling pathways and sequential events. Red lines with blunt ends indicate inhibition, downregulation, or targeted blockade. Small downward arrows adjacent to specific molecules denote a decrease in their expression levels.

Timosaponin A-III (Tim-AIII), a steroidal CHM bioactive component, was demonstrated by Zhou et al. [63] to induce ferroptosis by binding to heat-shock protein 90 (HSP90). This results in the formation of a Tim-AIII–HSP90 complex that targets, ubiquitinates, and degrades GPX4. In vivo assays further revealed that Tim-AIII not only significantly inhibits tumor growth but also improves the tumor microenvironment by reducing the proportion of regulatory T cells, demonstrating promising clinical potential. Furthermore, treatment of lung cancer cells with curcumenol leads to elevated intracellular ROS levels and depleted GSH levels, accompanied by altered expression of ferroptosis-related proteins. Notably, curcumenol-induced cell death is significantly rescued by ferroptosis inhibitors, whereas inhibitors of other cell death pathways show negligible effects. Further investigation identified that curcumenol modulates lncRNA H19 expression to trigger ferroptosis in lung cancer cells via the lncRNA H19/miR-19b-3p/FTH1 axis [64]. Erianin has also been shown to induce G2/M phase arrest and inhibit the proliferation and metastasis of lung cancer cells by regulating epithelial–mesenchymal transition (EMT)-related protein expression. Mechanistically, erianin promotes ferroptosis by activating the calcium (Ca^2+^)/calmodulin (CaM) signaling pathway; this is supported by the accumulation of ferroptotic markers and the effective rescue of cell death by ferroptosis inhibitors [65]. In A549 lung cancer cells and tumor tissues treated with 6-gingerol, a CHM bioactive component, increased numbers of autophagosomes were observed alongside elevated intracellular levels of ROS and Fe^2+^. These changes are accompanied by the upregulation of autophagy-related proteins, such as Beclin-1 and microtubule-associated protein 1 light chain 3 (MAP1LC3), and ferroptosis-related proteins like transferrin receptor 1 (TFR1), while the expression of ubiquitin-specific protease 14 (USP14) and FTH1 is downregulated. Subsequent experiments demonstrated that 6-gingerol promotes autophagy-dependent ferroptosis in lung cancer cells by inhibiting the expression of USP14 [66]. In a study involving A549 and H460 lung cancer cells, Wu et al. [67] performed an intervention utilizing dihydroisotanshinone I (DT), a CHM component derived from *Salvia miltiorrhiza*. They found and demonstrated that DT effectively suppresses cancer cell growth and viability by eliciting apoptosis and triggering GPX4 downregulation-mediated ferroptosis (Figure 3). Beyond its pro-ferroptotic effects in pancreatic cancer, solasonine (SS) exhibits potent anti-tumor activity against lung cancer. Mechanistically, SS disrupts the intracellular glutathione redox homeostasis, characterized by the depletion of GSH and cysteine (Cys) contents alongside the downregulation of GPX4 and SLC7A11 protein expression. Concomitantly, SS treatment induces mitochondrial membrane potential depolarization and augments mitochondrial ROS generation, indicating the involvement of mitochondrial impairment in SS-induced ferroptosis [68]. Similarly, artesunate and dihydroartemisinin (DHA) attenuate SLC7A11 expression at both the protein and mRNA levels while upregulating TFR1 mRNA in A549 cells. Notably, the growth inhibition of A549 cells was partially reversed by ferroptosis inhibitors and ROS scavengers, confirming that ART and DHA inhibit A549 cells by inducing ferroptosis through the regulation of ROS production [69]. Furthermore, DHA can be employed in combination therapy with gefitinib to reverse chemoresistance in NSCLC via the targeted accumulation of ROS [70]. While brusatol regulates downstream HO-1 through Nrf2 inhibition in GC cells [33], it activates the focal adhesion (FOCAD)/focal adhesion kinase (FAK) signaling pathway via Nrf2 inhibition in lung cancer cells, thereby sensitizing NSCLC cells to cysteine deprivation-induced ferroptosis [71]. Ophiopogonin B (OP-B), a saponin isolated from *Ophiopogon japonicus*, induces ferroptosis in NSCLC by suppressing the expression of the oncogene aurora kinase A (AURKA) [72]. This process is characterized by elevated levels of malondialdehyde (MDA), intracellular iron, and ROS, alongside decreased GSH levels, mitochondrial membrane potential, and altered protein expression [72]. Red ginseng polysaccharide (RGP) increases lactate dehydrogenase (LDH) release, promotes lipid ROS accumulation, and downregulates GPX4 expression in A549 cells [73]. The observation that GPX4 overexpression abolishes the anticancer effect of RGP suggests that RGP inhibits cancer cell growth by inducing GPX4 downregulation-mediated ferroptosis [73]. Following intervention in H460 and H1650 lung cancer cells, andrographolide (ADE), the primary CHM component of *Andrographis paniculata*, promotes LPO, MDA, and iron accumulation while inhibiting GSH levels and the expression of GPX4 and SLC7A11 [74]. Notably, pretreatment with the mitochondrial ROS scavenger Mito-TEMPO significantly attenuates these biochemical changes compared to ADE treatment alone, indicating that ADE-induced ferroptosis is mediated by mitochondrial dysfunction [74]. Sanguinarine (SAG) does not affect the mRNA levels of ferroptosis-related genes in lung cancer cells but markedly reduces GPX4 protein levels and shortens its half-life [75]. Mechanistically, SAG increases STIP1 homology and U-box containing protein 1 (STUB1) expression to promote the ubiquitin-mediated degradation of GPX4, thereby regulating STUB1/GPX4-mediated ferroptosis [75]. Qingrehuoxue formula (QRHXF) upregulates p53 and glycogen synthase kinase-3 beta (GSK-3β) (a serine/threonine kinase) while downregulating Nrf2 to elevate the levels of ROS, iron, H_2_O_2_, and MDA in tumor tissues. This process is accompanied by reduced GSH levels and the suppression of SLC7A11 and GPX4 expression, which ultimately induces ferroptosis [76]. *Hedyotis diffusa* (HD) has been confirmed to significantly inhibit the growth of various cancers [77] and is the primary constituent of *Hedyotis diffusa* injection (HDI). Distinct from other CHM bioactive components, the ferroptosis-inducing pathway of HDI does not involve the key target GPX4. Instead, HDI increases Bcl-2-associated X protein (Bax) expression by reducing the levels of the anti-apoptotic protein B-cell lymphoma 2 (Bcl2), which subsequently triggers the opening of voltage-dependent anion channels (VDAC) 2/3, leading to ROS accumulation and ferroptosis [78]. Similarly, the Xiaoyisanjie formula also downregulates Bcl2 and upregulates Bax. Specifically, components such as chrysin, apigenin, and kaempferol are considered key factors in inducing ferroptosis due to their strong affinity for GPX4. Furthermore, the Xiaoyisanjie formula inhibits GPX4 expression by upregulating miR-214-3p, providing another mechanism for ferroptosis induction [79]. Their detailed information is summarized in Table 6.

### 3.2. Nasopharyngeal Carcinoma

Cucurbitacin B inhibits the growth and metastasis of CNE1 NPC cells by inducing cell cycle arrest at the G2/M phase [80]. As a ferroptosis inducer, it primarily acts by downregulating GPX4 expression, reducing GSH production, and promoting iron accumulation [80]. Similarly, lupeol is characterized by reduced levels of GPX4 and GSH. Its mechanism involves increasing AMP-activated protein kinase alpha (AMPKα) phosphorylation and decreasing the levels of p-IκBα and nuclear factor kappa B (NF-κB) p65, which upregulates the expression of various ferroptosis markers via the AMPK/NF-κB pathway [81]. In contrast to lupeol, isoquercitrin inhibits AMPK activity, significantly reducing the p-AMPK/AMPK ratio. It also suppresses the activation of the NF-κB pathway, decreasing the p-p65/p65 and p-IκB/IκB ratios. Furthermore, isoquercitrin reduces the expression of activating transcription factor 4 (ATF4), SLC7A11, GPX4, and HO-1, thereby triggering ferroptosis through the inhibition of the AMPK/NF-κB pathway [82]. Moreover, allicin concurrently reduces GPX4 and GSH levels while decreasing the levels of lipid droplets that provide resistance to oxidative stress. This process is accompanied by increased lipid peroxidation, with these multiple mechanisms collectively inducing ferroptosis in NPC cells [83]. The pro-ferroptotic effect of luteolin can be reversed by SRY-box transcription factor 4 (SOX4) overexpression. Luteolin also inhibits SOX4 expression, which reduces the binding of SOX4 to the growth differentiation factor-15 (GDF15) promoter, thereby inducing ferroptosis. Although this study focused exclusively on the SOX4/GDF15 signaling pathway and did not explore other mechanisms, it highlights the potential of luteolin as a CHM component for NPC treatment [84]. Berberine (BBR) exhibits a strong interaction with GPX4. It not only downregulates GPX4 mRNA and protein levels via the System Xc−/GSH/GPX4 axis but also directly impairs GPX4 function, triggering intracellular oxidative stress to promote ferroptosis [85]. Finally, *Sophorae Tonkinensis* Radix et Rhizoma extract (TSRE) activates protein kinase R-like endoplasmic reticulum kinase (PERK) in CNE1 NPC cells. The resulting p-PERK activates the Nrf2/HO-1 signaling pathway to upregulate Nrf2 and HO-1 expression. This ultimately leads to ferroptotic characteristics in CNE1 cells, including elevated ROS, Fe^2+^, and lipid peroxidation levels, alongside decreased GSH levels [86]. In HNE1 NPC cells treated with phillyrin, a CHM bioactive component, the expression levels of miR-545-3p are significantly upregulated, while the expression levels of SLC7A11 are downregulated. Experimental results further confirmed that SLC7A11 is a direct target of miR-545-3p, suggesting that phillyrin induces ferroptosis via the miR-545-3p/SLC7A11 axis [87]. Pseudolaric acid B has also been found to promote ferroptosis. It induces a decrease in the levels of GSH and superoxide dismutase (SOD), along with reduced expression of GPX4, SLC7A11, and FTH1, while increasing MDA and ROS levels. However, its specific mechanism remains unclear and warrants further investigation [88]. Distinct from other CHM bioactive components, icaritin upregulates ACSL4 and downregulates GPX4 while significantly increasing the levels of ROS and phosphorylated H2AX (γ−H2AX). Since γ-H2AX is a marker of DNA breaks, this indicates that icaritin increases DNA damage in NPC cells and, through the induction of ferroptosis, enhances their radiosensitivity [89]. More detailed information on these compounds is provided in Table 7.

While CHM components show strong pro-ferroptotic activity in respiratory tumors cells, achieving effective therapeutic concentrations in vivo remains a challenge. This is largely due to the poor aqueous solubility and rapid metabolism of many hydrophobic monomers discussed above, such as andrographolide, erianin, and tanshinone derivatives [90]. However, agents with established safety profiles, such as DHA, exhibit clear clinical potential in reversing targeted therapy resistance [70].

## 4. Osteosarcoma

Osteosarcoma (OS) is the most common primary malignant bone tumor, predominantly occurring in the weight-bearing long bones of children and adolescents, such as the distal femur and proximal tibia [91]. Currently, the mainstay of clinical OS treatment remains combination therapy involving chemotherapy and surgery; however, chemotherapeutic drugs inevitably exhibit biological toxicity. Since neither increasing chemotherapy intensity nor long-term usage can adequately meet the survival needs of patients, the development of novel, low-toxicity drugs are essential [92]. Notably, researchers have found that certain CHM bioactive components can induce apoptosis in OS cells by modulating ferroptosis, suggesting significant potential for CHM bioactive components in the field of anti-osteosarcoma research.

Cinobufacini, an aqueous extract of dried toad skin, exhibits broad-spectrum antitumor efficacy. In U2OS cells, the significant upregulation of TFR1 and Nrf2 by cinobufacini augments intracellular iron and ROS levels. Concomitantly, the suppression of GPX4 expression elicits a robust oxidative stress response, ultimately driving ferroptosis in U2OS cells [93]. Curcumin has been confirmed to possess multi-target anticancer properties, modulating ferroptosis in OS cells via the Nrf2/GPX4 axis [94]. However, its application is hindered by poor stability and low intestinal absorption. In contrast, EF24 (3,5-bis[(2-fluorophenyl)methylene]-4-piperidinone), a curcumin analog, offers superior safety, bioavailability, killing selectivity, and broad-spectrum antitumor activity. Mechanistically, EF24 promotes HO-1 expression in U2OS and Saos-2 cells. Notably, the sensitization of both cell lines to EF24-induced ferroptosis is driven by HO-1 overexpression. Furthermore, elevated HO-1 expression can be detected in the tumor tissues of clinical OS patients, highlighting the translational potential of EF24 for clinical OS therapy [92]. The attenuation of U2OS proliferation by triptolide is accompanied by elevated intracellular Fe^2+^ and ROS levels. Mechanistically, this process is likely driven by the promotion of miR-34b-5p expression in U2OS cells, where the highly expressed miR-34b-5p targets Notch1 to elicit ferroptosis. However, since the specific role of Notch1 in ferroptosis was not investigated in this study, the precise modulatory function of the miR-34b-5p/Notch1 axis warrants further exploration [95]. Berberine, also known as huangliansu, exerts inhibitory effects against various tumors. In MG63 cells, BBR intervention elicits classical ferroptotic characteristics in mitochondria. Concomitantly, the augmentation of intracellular Fe^2+^, ROS, and MDA levels is accompanied by the depletion of GSH. Mechanistically, the downregulation of signal transducer and activator of transcription 3 (STAT3) promotes the upregulation of its downstream target, p53, thereby attenuating SLC7A11 levels. This suggests that BBR-induced ferroptosis in MG63 cells is driven by the STAT3/p53/SLC7A11 axis [96]. Notably, bavachin modulates this identical signaling pathway to induce ferroptosis [97]. Similarly, Li et al. discovered that artesunate upregulates p53 expression, which consequently attenuates the downstream expression of SLC7A11 and GPX4. Thus, the promotion of ferroptosis in U2OS cells by artesunate is contingent upon the p53/SLC7A11/GPX4 axis [98]. However, the potential modulation of STAT3 by artesunate—similar to berberine and bavachin—remains unverified. Consequently, further exploration upstream of this pathway is warranted. Sulforaphane (SFN) is a bioactive compound derived from cruciferous vegetables. Mechanistically, the direct binding of SFN to p62 augments the interaction between p62 and SLC7A11. The subsequent ferroptosis in OS cells is driven by the autolysosomal degradation of SLC7A11. Notably, the anticancer efficacy of SFN, accompanied by an absence of distinct toxicity, has been validated in vivo [99]. Theaflavin-3,3′-digallate (TF3) is an active component extracted from plants such as sumac, gallnut, and tea [100]. In MG63 and HOS cells treated with TF3, typical pro-ferroptotic morphological features are elicited in mitochondria. Concomitantly, the activation of the mitogen-activated protein kinase (MAPK) pathway is contingent upon TF3-triggered ROS generation. This activation elicits the phosphorylation of c-Jun N-terminal kinase (JNK), p38, and extracellular signal-regulated kinase (ERK), demonstrating excellent anticancer efficacy. Their detailed information is summarized in Table 8.

A principal limitation in osteosarcoma research is that the clinical efficacy of natural polyphenols, such as curcumin, is significantly hindered by their poor systemic bioavailability and chemical instability [101]. However, the development of synthetic analogs such as EF24, alongside the high-selectivity and low-toxicity profile of sulforaphane, successfully addresses these pharmacological bottlenecks, paving the way for safer systemic administration [92,99].

## 5. Breast Cancer

Globally, the incidence and mortality rates of breast cancer rank highest among all female malignancies, accounting for 15.4% of total cancer cases [21]. Clinically, the classification of this malignancy into three major subgroups—estrogen receptor-positive (ER+), human epidermal growth factor receptor 2-positive (HER2+), and triple-negative breast cancer (TNBC)—is contingent upon the expression profiles of estrogen receptors, progesterone receptors (PR), and human epidermal growth factor receptor 2. Among these subtypes, high rates of recurrence, aggressiveness, and metastasis distinctly characterize TNBC. Concomitantly, the poor prognosis of TNBC is driven by the emergence of drug resistance in advanced stages and the absence of precise therapeutic targets. Consequently, these combined factors elevate TNBC to the subgroup with the highest mortality rate, despite accounting for only approximately 15% of all breast cancer cases [102,103]. Therefore, growing academic interest has been directed toward CHM bioactive components. Mechanistically, the elicitation of ferroptosis in breast cancer cells by these components provides a novel perspective for the exploration of new therapeutic targets.

The extract of *Tripterygium wilfordii* attenuates the proliferation and migration of TNBC in a dose-dependent manner. Concomitantly, it exhibits the capacity to suppress breast cancer bone metastasis and reduce bone loss. Mechanistically, the elicitation of ferroptosis in 4T1 TNBC cells by this extract is driven by the downregulation of the Nrf2/SLC7A11/GPX4 pathway [104]. *Lycium barbarum* polysaccharide (LBP) exerts dose-dependent cytotoxicity, thereby attenuating the proliferation of MCF-7 and MDA-MB-231 breast cancer cells. Notably, pharmacological experiments utilizing the ferroptosis inhibitor Fer-1, the cystine/glutamate transporter (xCT) inhibitor erastin, and the GPX4 inhibitor RAS-selective lethal 3 (RSL3) demonstrate that LBP-induced ferroptosis is contingent upon the xCT/GPX4 signaling axis [105]. *Boswellia carterii* n-hexane extract (BCHE) has been confirmed to exhibit potent inhibitory effects against MCF-7 and MDA-MB-231 cells both in vitro and in vivo. Mechanistically, this efficacy is driven by the dual modulation of key ferroptotic pathways. Specifically, the upregulation of transferrin promotes intracellular Fe^2+^ accumulation, while the downregulation of GPX4 impairs the cellular capacity to clear lipid peroxides. Consequently, the excessive accumulation of ROS-mediated lipid peroxidation ultimately elicits ferroptosis in breast cancer cells [106]. Similarly, brazilin induces mitochondrial morphological damage in 4T1 cells. In vitro assays reveal that the dose-dependent attenuation of 4T1 cell viability, invasion, and migration by hematoxylin is driven by the induction of ferroptosis via the p53/SLC7A11/GPX4 signaling pathway [107]. Xihuang pill (XHP) demonstrates the impact of ubiquitination on ferroptosis. Mechanistically, the downregulation of the deubiquitinase OTUB1 by XHP inhibits OTUB1-mediated SLC7A11 deubiquitination. Consequently, the resulting acceleration of SLC7A11 proteasomal degradation attenuates GSH synthesis and GPX4 activity. Ultimately, the elicitation of ferroptosis by XHP effectively suppresses the proliferation and metastasis of TNBC cells [108]. At clinical concentrations, intervention with Shuganning injection (SGNI) selectively suppresses TNBC cell proliferation in vitro without affecting normal cells, while significantly inhibiting tumor growth in vivo. Mechanistically, the activation of the Nrf2/HO-1 signaling pathway by SGNI promotes the accumulation of the intracellular labile iron pool and lipid peroxidation. Consequently, this process selectively elicits ferroptosis in TNBC cells [109]. Similarly, the direct binding of saikosaponin D (SSD) to β-catenin in the Wnt pathway attenuates the high expression of its downstream target genes, c-Myc and cyclin D1, in TNBC cells, thereby suppressing cell proliferation [110]. The viability of MDA-MB-231 TNBC cells is dose-dependently inhibited by Yanghe decoction (YHD), a CHM formula with antitumor properties. Mechanistically, the initiation of autophagy by YHD is driven by the upregulation of peroxisome proliferator-activated receptor gamma (PPARγ) expression. Concomitantly, this autophagic process further promotes ferroptosis-related lipid peroxidation and Fe^2+^ accumulation, ultimately eliciting cellular ferroptosis [111]. Paclitaxel, a natural anticancer component extracted from yew bark, is a commonly used chemotherapeutic agent. Notably, the combination of paclitaxel with the ferroptosis inducer erastin exhibits a more potent anticancer efficacy than monotherapy. Mechanistically, the acceleration of the ferroptotic process by this combination therapy is driven by multiple events: the disruption of mitochondrial function, the exacerbation of oxidative stress and lipid peroxidation, the depletion of GSH levels, the attenuation of GPX activity, and the downregulation of ferroptosis-regulatory proteins. However, the specific targets and signaling pathways underlying this synergistic effect remain incompletely elucidated [112]. Oridonin (ORI) is the primary active component of the CHM *Rabdosia rubescens*. Mechanistically, the elicitation of cellular ferroptosis by ORI is driven by the suppression of GPX4. Concomitantly, ORI augments the anti-breast cancer efficacy of the ferroptosis inducer RAS-selective lethal 3 (RSL3) by inhibiting the JNK/Nrf2/HO-1 axis. Consequently, the elevation of ferroptosis-related indicators, such as ROS and Fe^2+^, along with mitochondrial damage, effectively attenuates breast cancer cell viability [113]. Danggui Buxue Tang (DBT) is a classic CHM formula. Studies demonstrate that DBT monotherapy attenuates the proliferation and viability of breast cancer cells. Notably, the cytotoxicity of doxorubicin (DOX) is significantly enhanced by its combinatory application with DBT. Mechanistically, this combined antitumor efficacy, which has been validated in vivo, is driven by the elicitation of ferroptosis. This process is characterized by the downregulation of proteins associated with the Nrf2/HO-1/GPX4 axis and the promotion of ROS generation. Furthermore, the combined application of DBT and DOX exhibits a unique hepatoprotective effect. Although the precise mechanism underlying this hepatoprotection remains unelucidated, it provides a novel perspective for the combinatory application of CHM bioactive components and chemotherapeutic agents [114]. A summary of these CHM bioactive components against breast cancer is presented in Table 9.

Notably, while crude extracts like *Tripterygium wilfordii* exhibit potent anti-TNBC effects, their clinical application is severely restricted by substantial hepatotoxicity and nephrotoxicity [115]. Therefore, focusing on highly purified monomers with defined safety profiles is better for clinical translation.

## 6. Hematological Malignancies

The clinical spectrum of hematological malignancies primarily encompasses leukemia, non-Hodgkin lymphoma, multiple myeloma, and Hodgkin lymphoma. Notably, based on Global Cancer 2022 data, the global incidence and annual mortality rates of these malignancies account for 6.56% and 7.19% of all cancer cases and cancer-related deaths, respectively. Among these, the relatively high incidence of non-Hodgkin lymphoma, leukemia, and multiple myeloma establishes them as primary focuses for clinical diagnosis and scientific exploration [21]. Currently, the primary clinical therapeutic modalities for hematological malignancies include chemotherapy, radiotherapy, immunotherapy, and bone marrow transplantation. However, the clinical efficacy of these conventional therapies is frequently attenuated by drug-induced toxicity and the emergence of tumor cell resistance. Consequently, these limitations inevitably exert adverse effects on patients’ quality of life [116,117]. Therefore, the urgent clinical demand necessitates the development of novel therapies or targeted agents. In this context, CHM bioactive components exhibit unique advantages, providing promising new strategies for the treatment of these malignancies [118].

### 6.1. Leukemia

Globally, the incidence and mortality rates of leukemia rank thirteenth and tenth, respectively, among all malignant diagnoses. In 2022, the global incidence of leukemia exceeded 487,000 new cases, with associated mortality reaching 305,000 deaths [21]. As a hematopoietic malignancy, the clinical categorization of leukemia comprises four primary subtypes. Notably, the manifestation of these subtypes exhibits distinct biological and epidemiological features. Specifically, the predominant occurrence of acute lymphoblastic leukemia (ALL) is observed in pediatric populations. Furthermore, the epidemiological distribution of ALL exhibits a bimodal trend, with a significant elevation in incidence rates observed in Latin American and Asian countries. Although acute myeloid leukemia (AML) is more common in adults, a relatively high incidence is also observed in children. Additionally, higher incidence rates of AML are reported in regions with a high Human Development Index (HDI). The predominant occurrence of chronic lymphocytic leukemia (CLL) is observed in elderly males. Furthermore, high incidence rates of CLL are reported in North America, Oceania, and certain European countries. Finally, a higher proportion of chronic myeloid leukemia (CML) cases is observed among adult males in high-HDI countries [119].

Dihydroartemisinin (DHA) modulates the AMPK/mTOR/p70S6k signaling pathway. Mechanistically, the autophagic degradation of FTH in AML cells is induced by DHA. Consequently, the acceleration of ferritin degradation promotes the accumulation of intracellular reactive oxygen species (ROS), ultimately eliciting ferroptosis in tumor cells [120]. Acetylshikonin is a naphthoquinone compound extracted from the traditional CHM *Lithospermum erythrorhizon*. Li et al. demonstrated that the promotion of ferroptosis in HL-60 cells by acetylshikonin involves dual mechanisms [121]. On the one hand, the upregulation of p53 expression reduces GSH levels, thereby inhibiting GPX4 expression and eliciting ferroptosis. On the other hand, the disruption of intracellular iron homeostasis in HL-60 cells is driven by the upregulation of CD71/TFR1 and the downregulation of FTH1, which further accelerates ferroptosis. Notably, these cellular alterations are reversed by the application of ferroptosis inhibitors. Realgar, initially recorded in the *Shennong Bencao Jing*, contains tetraarsenic tetrasulfide (As_4_S_4_) as its primary active component. Mechanistically, the elicitation of ferroptosis in B-cell acute lymphoblastic leukemia (B-ALL) cell lines by As_4_S_4_ is driven by the activation of the ROS/p53 axis [122]. Furthermore, Qinghuang powder, which features realgar as its principal component, has been widely applied in the clinical treatment of leukemia. Baicalein modulates the progression of AML. Mechanistically, this modulation is driven by the concentration-dependent accumulation of ROS. Specifically, at low concentrations, baicalein promotes ROS accumulation and myeloid cell differentiation, which is characterized by the upregulation of cluster of differentiation 11b (CD11b) and cluster of differentiation 14 (CD14) expression. Notably, this effect is reversed by ROS inhibitors. Conversely, at high concentrations, the elicitation of ferroptosis in tumor cells by baicalin is driven by the SLC7A11/GSH/GPX4 signaling pathway. Furthermore, this mechanism has been validated through molecular docking and SLC7A11 overexpression experiments [123]. Similarly, hesperadin effectively elicits ferroptosis in K562 chronic myeloid leukemia (CML) cells [124]. This pro-ferroptotic effect is characterized by a dose-dependent exacerbation of lipid peroxidation and a significant accumulation of the intracellular labile iron pool, which was experimentally confirmed using specific Fe^2+^ fluorescent probes [124]. A summary of these compounds is presented in Table 10.

### 6.2. Non-Hodgkin Lymphoma

In 2022, the global incidence of non-Hodgkin lymphoma (NHL) reached 553,000 new cases, with associated mortality encompassing 250,000 deaths. As the most prevalent hematological malignancy, NHL ranks as the tenth most frequently diagnosed cancer and the eleventh leading cause of cancer-related mortality globally [21]. Burkitt lymphoma (BL) is a highly aggressive malignancy driven by the malignant transformation of germinal center B cells [125]. Although high-intensity chemotherapy significantly improves the clinical prognosis for adults, achieving a 2-year overall survival rate of 60% to 70% [126], the survival rate among patients over 60 years of age remains disproportionately low [127,128]. Furthermore, mantle cell lymphoma (MCL) represents another highly aggressive and refractory subtype of B-cell non-Hodgkin lymphoma (B-NHL). Clinically characterized by a complex disease course and the lack of a curative standard treatment [129], MCL exhibits a 10-year survival rate of merely 5% to 10% and a median overall survival of only 3 years. Consequently, this represents the shortest survival duration among all NHL subtypes [130].

Wang et al. [131] demonstrated that artesunate induces ferroptosis in DAUDI and CA-46 cells, an effect validated by the protective interventions of liproxstatin-1 (Lip-1), ferrostatin-1 (Fer-1), and deferoxamine (DFO). This pro-ferroptotic process is accompanied by an endoplasmic reticulum stress response. Mechanistically, artesunate depletes intracellular GSH to attenuate ferroptosis resistance. This inhibition is contingent upon the activation of the ATF4-enhancer-binding protein homologous protein (CHOP)–glutathione-specific gamma-glutamylcyclotransferase 1 (CHAC1) pathway, which upregulates CHAC1 expression. Furthermore, in vivo mouse tumor xenograft models confirm that artesunate effectively attenuates CA-46 cell proliferation through the elicitation of ferroptosis. In vitro studies by Shen et al. [132] demonstrated that DAUDI cells treated with dehydroleucodine (DHL) exhibit typical ferroptotic morphology, a dose-dependent increase in lipid ROS, and a corresponding decrease in GSH levels. The reversal of DHL-induced cell death by Fer-1 further confirms its pro-ferroptotic efficacy. Mechanistically, DHL disrupts the antioxidant defense system in BL cells by downregulating SLC7A11, thereby rendering them highly sensitive to oxidative stress-induced ferroptosis. Although these findings highlight SLC7A11-mediated ferroptosis as a core anti-tumor mechanism of DHL, further in vivo investigations are warranted to fully elucidate the specific molecular pathways. Furthermore, Chen et al. [133] demonstrated that artesunate attenuates diffuse large B-cell lymphoma (DLBCL) cell proliferation by inducing apoptosis and cell cycle arrest. Additionally, artesunate effectively triggers ferroptosis in DLBCL cells by disrupting the STAT3 signaling pathway. Although hyperactivation of the phosphatidylinositol 3-kinase (PI3K) pathway establishes the PI3Kδ isoform as a crucial therapeutic target in MCL [134,135], the clinical efficacy of PI3Kδ inhibitors is hindered by acquired drug resistance [136,137]. Caffeic acid phenethyl ester (CAPE), a primary bioactive component of propolis, has emerged as a sensitizer [138]. Lu et al. [139] revealed that while the deletion of the chromobox homolog 5 (CBX5) gene drives PI3Kδ inhibitor resistance in MCL cells, CAPE treatment effectively upregulates CBX5 expression. Consequently, this CBX5 upregulation induces ferroptosis, thereby restoring the sensitivity of MCL cells to PI3Kδ inhibitors and effectively reversing drug resistance. A summary of these compounds is presented in Table 11.

### 6.3. Multiple Myeloma

In 2022, the global incidence of multiple myeloma (MM) reached 187,774 new cases, with associated mortality encompassing 121,252 deaths [21]. As a hematological malignancy driven by the abnormal proliferation of plasma cells, MM accounts for 10% to 15% of all hematological cancers and is responsible for 20% of hematology-related cancer deaths [140,141]. Notably, this disease predominantly affects the elderly population, and its clinical progression leads to the damage of key organs [142].

**Figure 4:**
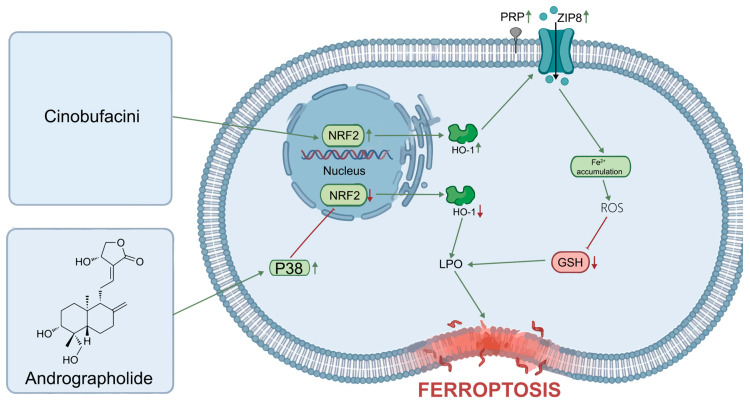
Schematic representation of CHM bioactive compounds inducing ferroptosis in MM cells. CHM bioactive components trigger ferroptosis through distinct regulatory effects on the Nrf2 network. Cinobufacini activates the Nrf2/HO-1 pathway and upregulates the PRP and ZIP8 transporters, driving Fe^2+^ and MDA accumulation, GSH depletion, and the elevation of ROS and intracellular PEGK levels. Conversely, andrographolide upregulates p38 to inhibit Nrf2, which subsequently downregulates HO-1 and promotes mitochondrial Fe^2+^ overload and LPO.

Cinobufacini effectively attenuates tumor growth by activating the Nrf2/HO-1 pathway. Concomitantly, the upregulation of PRP and ZIP8 expression promotes the accumulation of Fe^2+^ and MDA. Furthermore, this process induces the depletion of GSH levels alongside the elevation of intracellular ROS and PEGK levels. Consequently, the elicitation of ferroptosis in MM cells by cinobufacini has been firmly validated both in vitro and in vivo [143]. While cinobufacini promotes ferroptosis through Nrf2 activation, Li et al. [144] demonstrated that andrographolide utilizes a contrasting mechanism to trigger cell death in MM cell lines. Specifically, andrographolide upregulates p38 activity to downregulate Nrf2 expression, which subsequently inhibits the expression of HO-1. This signaling cascade leads to the elevation of intracellular and mitochondrial iron, as well as increased lipid peroxidation. Consequently, the targeted modulation of the p38/Nrf2/HO-1 pathway effectively drives ferroptotic cell death, a process that can be reversed by the application of ferroptosis inhibitors. Notably, both the hyperactivation and the targeted suppression of the Nrf2/HO-1 axis can lethally disrupt cellular iron and redox homeostasis to drive ferroptosis in specific pharmacological contexts, presenting a biologically consistent mechanism rather than a contradiction (Figure 4). A summary of these compounds is presented in Table 12.

Research on hematological malignancies is primarily limited to in vitro cell models. Among the evaluated agents, FDA-approved artemisinin derivatives like artesunate offer the most immediate clinical translation potential due to their well-documented human pharmacokinetics and minimal off-target toxicity compared to other experimental monomers [145].

## 7. Glioblastoma

Accounting for 50.9% of all primary malignant brain tumors, the incidence of glioblastoma (GBM) ranks highest among aggressive intracranial malignancies in adults [146]. Recent systematic analyses indicate that the global health burden of this disease is exacerbated by steadily climbing incidence rates across aging populations [21]. Despite comprehensive therapeutic interventions, such as maximal safe surgical resection, radiotherapy, and temozolomide (TMZ)-based chemotherapy, the prognosis of GBM remains extremely poor, with a 5-year relative survival rate of only 6.9% [146]. Such clinical outcomes are closely associated with the rapid emergence of acquired drug resistance and the unique anatomical constraints of the central nervous system, particularly the blood–brain barrier (BBB), which restricts the penetration of conventional chemotherapeutic agents [147]. Consequently, targeting ferroptosis is currently being investigated as a potential therapeutic strategy for GBM.

Recent studies have investigated several CHM bioactive components and their derivatives for their ability to modulate ferroptosis in glioblastoma cells. In temozolomide (TMZ)-resistant U87MG glioblastoma cells, the curcumin analog ALZ003 functions as a negative regulator of GPX4, decreasing the GSH/GSSG levels, thereby resulting in the accumulation of lipid peroxides and high ROS levels, culminating in ferroptosis [148]. Dihydroartemisinin initiates ferroptosis in U87 and A172 glioblastoma lines through the direct inhibition of GPX4, resulting in elevated ROS and lipid peroxidation [149]. Another artemisinin derivative, artesunate, has been shown to induce ferroptosis in glioblastoma cells both in vitro and in vivo through the regulation of iron metabolism and the modulation of p38 and ERK signaling pathways [150]. Capsaicin has also been reported to induce ferroptosis in U87-MG and U251 glioblastoma cells via the upregulation of ACSL4 and the downregulation of GPX4, thereby triggering a severe cellular redox imbalance [151]. Distinct from its modulation of hepatocellular carcinoma through AMPK-activated autophagy and ferroptosis regulation, brucine initiates ferroptosis in glioblastoma cells via ATF3 upregulation and the subsequent accumulation of hydrogen peroxide and iron [152]. A summary of these CHM bioactive components against glioblastoma via ferroptosis is presented in Table 13.

While the aforementioned CHM bioactive components show ferroptotic efficacy in preclinical models (such as the U87, U251, and A172 cell lines), their in vivo efficacy and clinical translation are largely restricted by the BBB [147]. Furthermore, despite the scientific value of these in vitro and in vivo models, they exhibit inherent limitations. Specifically, traditional in vitro cultures are not favorable to maintain various cellular subtypes, while xenograft models exhibit variable engraftment efficiency and host infiltration, and have very limited throughput [153]. Additionally, since ALZ003 has already shown efficacy in TMZ-resistant cells [148], exploring the synergistic application of these CHM components with standard-of-care treatments represents a highly translational strategy.

## 8. Discussion

**Figure 5:**
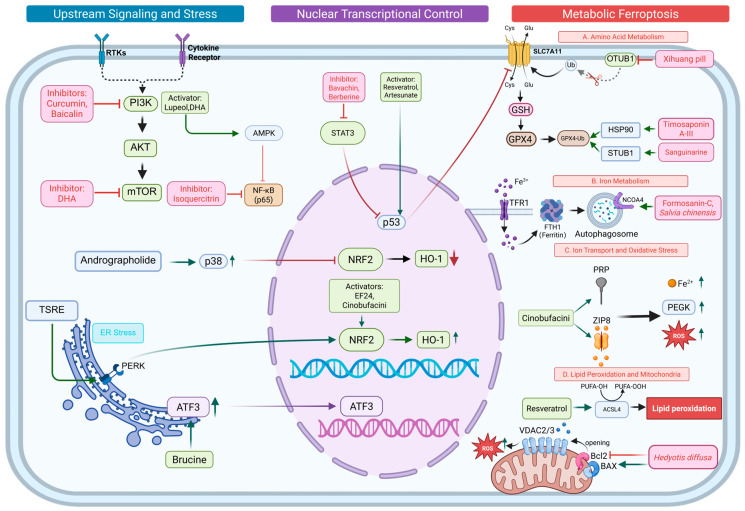
Integrative schematic summarizing the principal mechanisms and main regulatory nodes through which bioactive components of Chinese herbal medicine converge to induce ferroptosis in cancer cells. As illustrated, the mechanism is organized into three main sections: (1) Upstream Signaling and Stress, (2) Nuclear Transcriptional Control, and (3) Metabolic Ferroptosis. In the upstream signaling phase, diverse intracellular cascades (e.g., PI3K/AKT/mTOR and AMPK/NF-κB) and ER stress pathways are modulated by specific bioactive components to transduce apoptotic and stress signals. Within the nucleus, these diverse signaling pathways converge on key transcription factors. Specifically, p53 is upregulated to repress SLC7A11 transcription. Concurrently, NRF2 undergoes context-dependent bidirectional regulation (targeted activation or suppression) to disrupt intracellular iron and redox homeostasis via HO-1, while ATF3 is upregulated following ER stress. The core metabolic execution of ferroptosis encompasses four distinct molecular nodes: (A) Amino Acid Metabolism: Blockade of the System Xc-/GSH/GPX4 axis and the ubiquitin-proteasome-dependent degradation of GPX4 and SLC7A11; (B) Iron Metabolism: NCOA4-mediated ferritinophagy promotes iron overload; (C) Ion Transport and Oxidative Stress: Upregulation of ion transporters (PRP, ZIP8) accelerates Fe^2+^ and ROS accumulation; and (D) Lipid Peroxidation and Mitochondria: ACSL4 drives lethal lipid peroxidation, while the modulation of the BAX/Bcl2 axis triggers VDAC2/3 opening, leading to mitochondrial membrane permeabilization. In the diagram, solid arrows indicate promotion, activation, or downstream signaling; T-bar lines represent pharmacological inhibition or transcriptional blockade; dashed arrows denote indirect regulation or multi-step pathways; and upward and downward arrows adjacent to specific molecules signify their upregulation and downregulation, respectively, induced by the corresponding CHM bioactive components.

This review focuses on the regulatory role of CHM bioactive components in ferroptosis across common cancers, systematically reviewing research findings related to gastrointestinal tumors, respiratory malignancies, breast cancer, osteosarcoma, hematological malignancies, and glioblastoma. In summary, the key targets are primarily concentrated on GPX4, SLC7A11, p53, and Nrf2. Among these, GPX4 and SLC7A11, acting as crucial core molecules inhibiting ferroptosis, serve as the primary targets for most CHM bioactive components. Specifically, the downregulation of GPX4 and SLC7A11 expression by CHM bioactive components inhibits their activity, thereby triggering lipid peroxidation and iron accumulation. Furthermore, this overarching regulation is mediated by the specific activation or inhibition of multiple signaling cascades, such as the PI3K/AKT/mTOR, AMPK/NF-κB, and p53/SLC7A11/GPX4 pathways (Figure 5). Clinically, these components exhibit diverse effects across different cancer types. In gastrointestinal tumors, components like honokiol and baicalin exhibit broad anti-tumor efficacy. However, their specific mechanisms exhibit cell-type specificity. Concomitantly, certain CHM formulas prevent hepatocarcinogenesis through the protection of normal cells, which breaks through the traditional paradigm of merely inducing tumor cell death. In respiratory malignancies, CHM bioactive components effectively elicit ferroptosis. Crucially, the reversal of chemoresistance to EGFR-TKIs and gefitinib is induced by these agents. Additionally, for specific components like solamargine, the promotion of ferroptosis is driven by mitochondrial damage. In osteosarcoma, components such as curcumin analogs and sulforaphane demonstrate low toxicity and high selectivity. Mechanistically, their anti-tumor efficacy is driven by the modulation of the STAT3/p53/SLC7A11 and MAPK pathways. Therefore, this provides a novel strategy to mitigate chemotherapy-induced toxicity.

Despite these promising prospects, the clinical translation of CHM bioactive components faces several bottlenecks. Primarily, due to the complexity of herbal extracts, the establishment of standardized quality control systems for active component purity remains inadequate. Additionally, current research predominantly focuses on qualitative mechanistic explorations. Consequently, the determination of precise in vivo quantitative analyses, pharmacokinetic profiles, and optimal effective doses for CHM monomers or formulas remains elusive. Most crucially, the current understanding of CHM-induced ferroptosis is largely restricted to in vitro cell lines and in vivo animal models. Therefore, the validation of authentic clinical efficacy and long-term safety is severely limited by the absence of large-scale clinical trials. Nevertheless, when evaluating which components are supported by the strongest evidence and show the greatest promise for clinical translation, artemisinin derivatives (such as artesunate and DHA) emerge as the premier candidates. As clinically established antimalarial drugs, their safety profiles and human pharmacokinetics are already well-documented. Additionally, structurally optimized agents like curcumin analog ALZ003 demonstrate profound translational value, particularly through their synergistic ability to reverse multidrug resistance when combined with conventional chemotherapies. Moving forward, future research must elucidate the crosstalk mechanisms between ferroptosis and other cell death modalities, such as apoptosis and autophagy to facilitate the construction of a comprehensive multi-target anticancer network. Furthermore, the mitigation of poor water solubility and low absorption rates associated with monomers like curcumin requires the improvement of targeted drug delivery systems, such as nanotechnology and liposomes, to achieve precise targeted delivery. Ultimately, given that the reversal of traditional therapeutic resistance is driven by ferroptosis, the exploration of combinatorial regimens involving CHM and established first-line anticancer drugs represents a highly promising research direction.

## Figures and Tables

**Table 1:** Summary of CHM bioactive components against colorectal cancer via ferroptosis.

Type of Material	Name	Modulation of Ferroptosis	Cell Line(s)	Reference
Pure Compound	Resveratrol	Upregulation of ACSL4 and p53; Downregulation of GPX4 and FTH	HCT116	[23]
Extract	β-elemene	Upregulation of HO-1; Downregulation of GPX4, SLC7A11, and FTH	HCT116 (KRAS mutant), LoVo (KRAS mutant), Caco-2 (KRAS wild-type)	[24]
Pure Compound	Honokiol	Downregulation of GPX4	RKO, HCT116, SW48, HT29, LS174T, HCT8, SW480	[25]
Extract	Methanolic extract of *B. etnensis* Raf. bark	Upregulation of HO-1	Caco-2	[26]

**Table 2:** Summary of CHM bioactive components compounds against GC via ferroptosis.

Type of Material	Name	Modulation of Ferroptosis	Cell Line(s)/Model	Reference
Pure Compound	Honokiol	Downregulation of GPX4, p-YAP1, and YAP1	GC mouse model	[27]
Pure Compound	Ophiopogonin B	Downregulation of GPX4 and SLC7A11	AGS, NCI-N87	[28]
Pure Compound	Curcumin	Downregulation of GPX4 and SLC7A11; Inactivation of PI3K/AKT/mTOR pathway	AGS, HGC-27	[29]
Pure Compound	Tanshinone IIA	Upregulation of PTGS2, CHAC1, and p53; Downregulation of SLC7A11	BGC-823, NCI-H87	[30]
Pure Compound	Baicalin	Upregulation of p53; Downregulation of SLC7A11	SGC-7901	[31]
Pure Compound	Brusatol	Inhibition of Nrf2/HO-1 pathway	HGC-27	[33]
Pure Compound	a2	Downregulation of GPX4	MGC-803, HGC-27, SGC-7901, MKN-45, AGS, BGC-823	[34]

**Table 3:** Summary of CHM bioactive components against liver cancer via ferroptosis.

Type of Material	Name	Modulation of Ferroptosis	Cell Line(s)/Model	Reference
Pure Compound	Brucine	Activation of AMPK; Inhibition of mTOR	Hep3B, HepG2, Huh7, HL-7702	[35]
Extract	*Scutellaria barbata*	Upregulation of IREB2 and ACSL4; Downregulation of GPX4 and SLC7A11	SMMC-7721, HepG2, Huh7	[36]
Pure Compound	Emodin	Upregulation of miR-1226-3p; Downregulation of GPX4	HepG2, LM3, and Hep3B	[37]
Pure Compound	Baicalin	Inhibition of PI3K/AKT/FOXO3a pathway	HepG2	[39]
Pure Compound	Formosanin-C	Upregulation of NCOA4; Downregulation of FTH	Hep3B, HepG2	[40]
Pure Compound	Icariin	Downregulation of GPX4, SLC7A11, PPARG, and FABP4	HepG2	[41]
Pure Compound	Cryptotanshinone	Downregulation of GPX4 and SLC7A11	HepG2	[42]
Formula	Huayu Qutan Formula	Upregulation of GPX4 and SLC7A11; Downregulation of p53	High-fat diet-induced liver cancer mouse model	[44]

**Table 4:** Summary of CHM bioactive components against pancreatic cancer via ferroptosis.

Type of Material	Name	Modulation of Ferroptosis	Cell Line(s)	Reference
Pure Compound	Celastrol	Downregulation of GPX4	PANC-1	[45]
Pure Compound	Wogonin	Downregulation of Nrf2 and GPX4	PANC-1, AsPC-1	[47]
Pure Compound	Solasonine	Downregulation of TFAP2A, OTUB1, and SLC7A11	PANC-1, CFPAC-1	[48]
Pure Compound	Ponicidin	Downregulation of GPX4	SW1990	[49]

**Table 5:** Summary of CHM bioactive components against Esophageal cancer via ferroptosis.

Type of Material	Name	Modulation of Ferroptosis	Cell Line(s)/Model	Reference
Pure Compound	Ferulic acid	Upregulation of ACSL4 and HO-1; Downregulation of GPX4 and SLC7A11	TE-4, EC-1	[51]
Pure Compound	Trigonelline	Inhibition of Nrf2/SLC7A11/GPX4 pathway	KYSE-150	[52]
Formula	Huqi Powder	Upregulation of p53; Downregulation of GPX4	EC9706	[53]
Extract	*Salvia chinensis*	Upregulation of NCOA4; Downregulation of GPX4	Esophageal cancer mouse model	[54]
Pure Compound	Artesunate	Downregulation of FTH	KYSE-150	[55]
Pure Compound	Resveratrol	Upregulation of ACSL4 and p53; Downregulation of FTH and GPX4	Eca109, Eca109/DDP	[56]

**Table 6:** Summary of CHM bioactive components against lung cancer via ferroptosis.

Type of Material	Name	Modulation of Ferroptosis	Cell Line(s)/Model	Reference
Pure Compound	Timosaponin A-III	Binding to HSP90; Downregulation of GPX4	H1299, A549, SPC-A1, LLC	[63]
Pure Compound	Curcumenol	Downregulation of lncRNA H19	H1299, H460	[64]
Pure Compound	Erianin	Activation of Ca^2+^/CaM	H1299, H460	[65]
Pure Compound	6-Gingerol	Upregulation of Beclin-1, MAP1LC3, and TFR1; Downregulation of USP14 and FTH1	A549	[66]
Pure Compound	Dihydroisotanshinone I	Downregulation of GPX4	A549, H460	[67]
Pure Compound	Solasonine	Downregulation of GPX4 and SLC7A11	A549, Calu-1	[68]
Pure Compound	Artesunate and dihydroartemisinin	Upregulation of TFR1; Downregulation of SLC7A11	A549	[69]
Pure Compound	Brusatol	Downregulation of Nrf2; Activation of FOCAD/FAK	A549, HCC827, H1975	[71]
Pure Compound	Ophiopogonin B	Inhibition of AURKA	A549	[72]
Extract	Red ginseng polysaccharide	Downregulation of GPX4	A549	[73]
Pure Compound	Andrographolide	Downregulation of GPX4 and SLC7A11	H460, H1650	[74]
Pure Compound	Sanguinarine	Downregulation of GPX4	A549, H3122	[75]
Formula	Qingrehuoxue formula	Upregulation of p53 and GSK-3β; Downregulation of Nrf2	Nude mouse model	[76]
Extract	Hedyotis diffusa injection	Activation of VDAC2/3	A549, H1975, nude mouse model	[78]
Formula	Xiaoyisanjie formula	Downregulation of BCL2; Upregulation of BAX and miR-214-3p	A549, PC9	[79]

**Table 7:** Summary of CHM bioactive components against NPC via ferroptosis.

Type of Material	Name	Modulation of Ferroptosis	Cell Line(s)/Model	Reference
Pure Compound	Cucurbitacin B	Downregulation of GPX4	CNE1	[80]
Pure Compound	Lupeol	Increased phosphorylation of AMPKα; Decreased p-IκBα and NF-κB p65	CNE1, 5-8F	[81]
Pure Compound	Isoquercitrin	Inhibition of AMPK/NF-κB pathway	CNE1, HNE1	[82]
Pure Compound	Allicin	Downregulation of GPX4 and GSH	HONE1, HNE1	[83]
Pure Compound	Luteolin	Inhibition of SOX4	NPC53, HNE3	[84]
Pure Compound	Berberine	Inhibition of System Xc−/GSH/GPX4 axis	S18, 5-8F, nude mouse model	[85]
Extract	TSRE	Activation of PERK	CNE1	[86]
Pure Compound	Phillyrin	Upregulation of miR-545-3p; Inhibition of SLC7A11	HNE1	[87]
Pure Compound	Pseudolaric acid B	Not elucidated	CNE1	[88]
Pure Compound	Icaritin	Increased DNA damage	HONE1, HNE1	[89]

**Table 8:** Summary of CHM bioactive components against osteosarcoma via ferroptosis.

Type of Material	Name	Modulation of Ferroptosis	Cell Line(s)/Model	Reference
Extract	Cinobufacini	Upregulation of TFR1 and Nrf2; Downregulation of GPX4	U2OS	[93]
Pure Compound	EF24	Upregulation of HO-1	U2OS, Saos-2	[92]
Pure Compound	Triptolide	Upregulation of miR-34b-5p	U2OS	[95]
Pure Compound	Berberine	Downregulation of STAT3	MG63	[96]
Pure Compound	Bavachin	Downregulation of STAT3	MG63, HOS	[97]
Pure Compound	Artesunate	Upregulation of p53	U2OS	[98]
Pure Compound	Sulforaphane	Binding to p62	143B, SJSA-1, nude mouse model	[99]
Pure Compound	TF3	Activation of MAPK	MG63, HOS	[100]

**Table 9:** Summary of CHM bioactive components against breast cancer via ferroptosis.

Type of Material	Name	Modulation of Ferroptosis	Cell Line(s)/Model	Reference
Extract	*Tripterygium* *wilfordii*	Inhibition of Nrf2/SLC7A11/ GPX4 pathway	4T1	[104]
Extract	*Lycium barbarum* polysaccharide	Inhibition of xCT/GPX4 axis	MCF-7, MDA-MB-231	[105]
Extract	BCHE	Upregulation of transferrin; Downregulation of GPX4	MCF-7, MDA-MB-231	[106]
Pure Compound	Brazilin	Inhibition of p53/SLC7A11/ GPX4 pathway	4T1	[107]
Formula	Xihuang pill	Downregulation of OTUB1	MDA-MB-231, SUM159PT, MCF-7	[108]
Formula	Shuganning injection	Activation of Nrf2/HO-1 pathway	MDA-MB-231, MDA-MB-468, BT-549	[109]
Pure Compound	Saikosaponin D	Inhibition of β-catenin	HCC1937, MDA-MB-231, MDA-MB-468, SUM159	[110]
Formula	Yanghe decoction	Upregulation of PPARγ	MDA-MB-231	[111]
Pure Compound	Paclitaxel	Incompletely elucidated	MCF-7, MDA-MB-231, MCF-10A	[112]
Pure Compound	Oridonin	Inhibition of JNK/Nrf2/HO-1 pathway	MCF-7, MDA-MB-231	[113]
Formula	Danggui Buxue Tang	Inhibition of Nrf2/HO-1/GPX4 pathway	4T1, MDA-MB-231, MCF-10A	[114]

**Table 10:** Summary of CHM bioactive components against Leukemia via ferroptosis.

Type of Material	Name	Modulation of Ferroptosis	Cell Line(s)/Model	Reference
Pure Compound	Dihydroartemisinin	Promotion of AMPK phosphorylation; Downregulation of mTOR/p70S6K pathway	HL-60, KG-1, THP-1	[120]
Pure Compound	Acetylshikonin	Upregulation of p53 and TFR1; Downregulation of GPX4 and FTH1	HL-60	[121]
Pure Compound	Tetraarsenic tetrasulfide	Upregulation of p53; Downregulation of GPX4 and SLC7A11	Human B-ALL cell lines, UCMSCs, 293T	[122]
Pure Compound	Baicalein	Downregulation of SLC7A11/GSH/ GPX4 pathway	HL-60	[123]
Pure Compound	Hesperadin	Downregulation of SLC7A11 and GPX4	K562	[124]

**Table 11:** Summary of CHM bioactive components against non-hodgkin lymphoma via ferroptosis.

Type of Material	Name	Modulation of Ferroptosis	Cell Line(s)/Model	Reference
Pure Compound	Artesunate	Activation of ATF4/CHOP/ CHAC1 pathway; Upregulation of CHAC1	Daudi, CA46, nude mouse model	[131]
Pure Compound	DHL	Downregulation of GSH and SLC7A11	Daudi	[132]
Pure Compound	Artesunate	Inhibition of STAT3 pathway	DLBCL	[133]
Pure Compound	Caffeic acid phenethyl ester	Upregulation of CBX5	MINO, JeKo-1	[139]

**Table 12:** Summary of CHM bioactive components against multiple myeloma via ferroptosis.

Type of Material	Name	Modulation of Ferroptosis	Cell Line(s)/Model	Reference
Extract	Cinobufacini	Upregulation of PRP and ZIP8; Downregulation of GSH	ARP-1, CAG, JJN3	[143]
Pure Compound	Andrographolide	Upregulation of p38; Downregulation of Nrf2 and HO-1	RPMI 8226, U266	[144]

**Table 13:** Summary of CHM bioactive components against glioblastoma via ferroptosis.

Type of Material	Name	Modulation of Ferroptosis	Cell Line(s)/Model	Reference
Pure Compound	ALZ003	Downregulation of GPX4; Decrease in GSH/GSSG levels	U87MG (TMZ- resistant)	[148]
Pure Compound	Dihydroartemisinin	Downregulation of GPX4	U87, A172	[149]
Pure Compound	Artesunate	Regulation of iron metabolism; Modulation of p38 and ERK signaling pathways	U251, U251 xenografts	[150]
Pure Compound	Capsaicin	Upregulation of ACSL4; Downregulation of GPX4	U87-MG, U251	[151]
Pure Compound	Brucine	Upregulation of ATF3; Accumulation of H_2_O_2_ and iron	U118, U87, U251, A172; U87 xenografts	[152]

## Data Availability

No new data were created or analyzed in this study. Data sharing is not applicable to this article.

## Abbreviations

The following abbreviations are used in this manuscript:

5-FU	5-fluorouracil
ACSL4	Acyl-CoA synthetase long-chain family member 4
ADE	Andrographolide
AKR1C3	Aldo-keto reductase family 1 member C3
ALL	Acute lymphoblastic leukemia
AML	Acute myeloid leukemia
AMPK	Adenosine monophosphate-activated protein kinase
AMPKα	AMP-activated protein kinase alpha
As_4_S_4_	Tetraarsenic tetrasulfide
ATF4	Activating transcription factor 4
AURKA	Aurora kinase A
B-ALL	B-cell acute lymphoblastic leukemia
B-NHL	B-cell non-Hodgkin lymphoma
Bax	Bcl-2-associated X protein
BBB	Blood–brain barrier
BBR	Berberine
BCHE	Boswellia carterii n-hexane extract
Bcl2	B-cell lymphoma 2
BL	Burkitt lymphoma
CaM	Calmodulin
CAPE	Caffeic acid phenethyl ester
CBX5	Chromobox homolog 5
CD11b	Cluster of differentiation 11b
CD14	Cluster of differentiation 14
Cel	Celastrol
CHAC1	Glutathione-specific gamma-glutamylcyclotransferase 1
CHM	Chinese herbal medicine
CHOP	Enhancer-binding protein homologous protein
CLL	Chronic lymphocytic leukemia
CML	Chronic myeloid leukemia
CRC	Colorectal cancer
Cys	Cysteine
DBT	Danggui Buxue Tang
DFO	Deferoxamine
DHA	Dihydroartemisinin
DHL	Dehydroleucodine
DLBCL	Diffuse large B-cell lymphoma
DOX	Doxorubicin
DT	Dihydroisotanshinone I
EF24	3,5-bis[(2-fluorophenyl)methylene]-4-piperidinone
EMT	Epithelial–mesenchymal transition
ER+	Estrogen receptor-positive
ERK	Extracellular signal-regulated kinase
ESCC	Esophageal squamous cell carcinoma
FA	Ferulic acid
FABP4	Fatty acid-binding protein 4
FAK	Focal adhesion kinase
FC	Formosanin-C
Fer-1	Ferrostatin-1
FOCAD	Focal adhesion
FTH	Ferritin heavy chain
GBM	Glioblastoma
GC	Gastric cancer
GDF15	Growth differentiation factor-15
GI	Gastrointestinal
GPX4	Glutathione peroxidase 4
GSH	Glutathione
GSK-3β	Glycogen synthase kinase-3 beta
HCC	Hepatocellular carcinoma
HD	Hedyotis diffusa
HDI	Hedyotis diffusa injection/Human Development Index
HER2+	Human epidermal growth factor receptor 2-positive
HO-1	Heme oxygenase-1
HSP90	Heat-shock protein 90
IREB2	Iron-responsive element binding protein 2
JNK	c-Jun N-terminal kinase
LBP	Lycium barbarum polysaccharide
LDH	Lactate dehydrogenase
Lip-1	Liproxstatin-1
LPO	Lipid peroxidation
MAP1LC3	Microtubule-associated protein 1 light chain 3
MAPK	Mitogen-activated protein kinase
MCL	Mantle cell lymphoma
MDA	Malondialdehyde
MDR	Multidrug resistance
MM	Multiple myeloma
NCOA4	Nuclear receptor coactivator 4
NF-κB	Nuclear factor kappa B
NHL	Non-Hodgkin lymphoma
NPC	Nasopharyngeal carcinoma
Nrf2	Nuclear factor erythroid-2-related factor 2
NSCLC	Non-small cell lung cancer
OP-B	Ophiopogonin B
ORI	Oridonin
OS	Osteosarcoma
PERK	Protein kinase R-like endoplasmic reticulum kinase
PI3K	Phosphatidylinositol 3-kinase
PPARγ/PPARG	Peroxisome proliferator-activated receptor gamma
PR	Progesterone receptors
PTGS2	Prostaglandin-endoperoxide synthase 2
PUFA	Polyunsaturated fatty acid
QRHXF	Qingrehuoxue formula
RGP	Red ginseng polysaccharide
ROS	Reactive oxygen species
RSL3	RAS-selective lethal 3
SAG	Sanguinarine
SFN	Sulforaphane
SGNI	Shuganning injection
SLC7A11	Solute carrier family 7 member 11
SOD	Superoxide dismutase
SOX4	SRY-box transcription factor 4
SS	Solasonine
SSD	Saikosaponin D
STAT3	Signal transducer and activator of transcription 3
STUB1	STIP1 homology and U-box containing protein 1
Tan IIA	Tanshinone IIA
TF3	Theaflavin-3,3′-digallate
TFAP2A	Transcription factor AP-2 alpha
TFR1	Transferrin receptor 1
Tim-AIII	Timosaponin A-III
TME	Tumor microenvironment
TMZ	Temozolomide
TNBC	Triple-negative breast cancer
TSRE	Sophorae Tonkinensis Radix et Rhizoma extract
USP14	Ubiquitin-specific protease 14
VDAC	Voltage-dependent anion channels
xCT	Cystine/glutamate transporter
XHP	Xihuang pill
YAP	Yes-associated protein
YHD	Yanghe decoction
γ-H2AX	Phosphorylated H2AX
